# Femtosecond Laser Microfabrication of Porous Superwetting Materials for Oil/Water Separation: A Mini-Review

**DOI:** 10.3389/fchem.2020.585723

**Published:** 2020-09-25

**Authors:** Na Feng, Jiale Yong

**Affiliations:** ^1^School of Chemistry and Chemical Engineering, Southwest University, Chongqing, China; ^2^School of Electronic Science and Engineering, Xi'an Jiaotong University, Xi'an, China

**Keywords:** oil/water separation, femtosecond laser, superhydrophobicity, underwater superoleophobicity, superwetting, porous material

## Abstract

Frequent oil-leakage accidents and large quantities of oil-bearing wastewater discharge cause severe environmental pollution and huge economic losses. Recently, superwetting porous materials are successfully utilized to separate oil/water mixture (OWM) based on the different interfacial behavior of water and oil. Here, we summarize the recent development of efficient oil/water separation (OWS) based on the femtosecond laser-induced superwetting materials. The typical wettability-based separation manners (including “oil-removing” and “water-removing”) and the characteristic of the femtosecond laser are introduced as background. Various laser-structured porous sheets with either superhydrophobicity or underwater superoleophobicity are successfully used to separate different OWMs. The laser processing methods, surface wettability, separation process, and separation mechanism of these laser-structured separation materials are reviewed. Finally, the current challenges and prospects in achieving OWS by femtosecond laser microfabrication are discussed.

## Introduction

Energy plays a vital role in human life. However, the most widely used petrochemical energy causes a series of environmental pollution problems. With the continuous growth of global energy demand, oil leakage accidents frequently occur, and a large volume of industrial oily wastewater is discharged, leading to serious ecological and environmental problems (Xue et al., 2014; Chu et al., 2015; Wang et al., 2015; Yong et al., 2016a; Gupta et al., 2017; Yong J. et al., 2018b). In 1989, the “Valdez” oil tanker spilled 11 million gallons of oil into the ocean near the Prince William Sound (Alaska) after running on rocks^1^. In 2002, the “Prestige” tanker sank in a storm, dumping 20 million gallons of fuel oil off the coast of Spain^2^. In 2010, the famous Gulf of Mexico oil spill leaked 210 million gallons of crude oil to the sea^3^. Such distressing accidents have occurred frequently. Besides, many industrial productions produce a large number of oily sewages daily, such as food production, metal smelting, textile industry, mining, biological pharmaceutical, petrochemical products, and so on (Gupta et al., 2017). This oily sewage becomes severe pollutants around the world. Frequent oil-leakage accidents and large quantities of oil-bearing discharge have caused a series of environmental damage/pollution and enormous economic loss. Many creatures that lived in the polluted waters died off or even came close to extinction. Toxic mixtures (such as hydrogen sulfide, toluene, and aromatic hydrocarbons) in oil spills can quickly enter marine ecosystems and the food chain, resulting in long-term harm to not only lower algae but also higher mammals. To deal with these serious pollution problems of marine crude oil and industrial waste oil, many technologies and materials have been carried out to perform efficient oil/water separation (OWS) (Xue et al., 2014; Chu et al., 2015; Wang et al., 2015; Yong et al., 2016a; Gupta et al., 2017; Yong J. et al., 2018b). The traditional separation methods include adsorption, gravity separation, flotation, skimming, centrifugal separation, etc. (Xue et al., 2014; Wang et al., 2015; Yong J. et al., 2018b). Although these methods can handle most oil/water mixtures (OWMs) to a certain extent, many limitations still exist, such as the requirement of input-driven energy, low separation efficiency, secondary pollution, and so on (Xue et al., 2014; Wang et al., 2015; Yong J. et al., 2018b). These limitations have led to the continuous development of more efficient and environmentally friendly OWS materials and systems.

Recently, superwetting porous materials are applied in OWS according to the different interfacial behavior of water and oil (Xue et al., 2014; Chu et al., 2015; Wang et al., 2015; Yong et al., 2016a; Gupta et al., 2017; Yong J. et al., 2018b). Those materials usually have opposite superwetting behaviors to water and oil, respectively, i.e., superhydrophobicity/superoleophilicity or superoleophobicity/superhydrophilicity (Tao et al., 2014; Zhu and Pan, 2014; Kong et al., 2015; Liu et al., 2015; Xue et al., 2015; Li J. et al., 2016; Su et al., 2018; Wang et al., 2018). Since femtosecond laser has many special features in preparing superwetting materials, a variety of OWS materials with superhydrophobicity or underwater superoleophobicity have been fabricated by femtosecond laser processing.

In this paper, the applications of the femtosecond laser-structured superwetting materials in OWS are reviewed. Firstly, the significance and urgency of performing efficient OWS are highlighted as the background. Secondly, we briefly introduce two typical manners (including “oil-removing” and “water-removing”) to separate OWMs based on the porous superwetting materials and the features of the femtosecond laser. Subsequently, we summarize the femtosecond laser-structured superwetting porous sheets/membranes that can separate various OWMs, mainly focusing on the laser processing method, surface wettability, separation process, and separation mechanism. Finally, the current challenges and prospects in achieving OWS by femtosecond laser microfabrication are discussed.

## Oil/Water Separation Based on the Superwetting Materials

**Figure 1:**
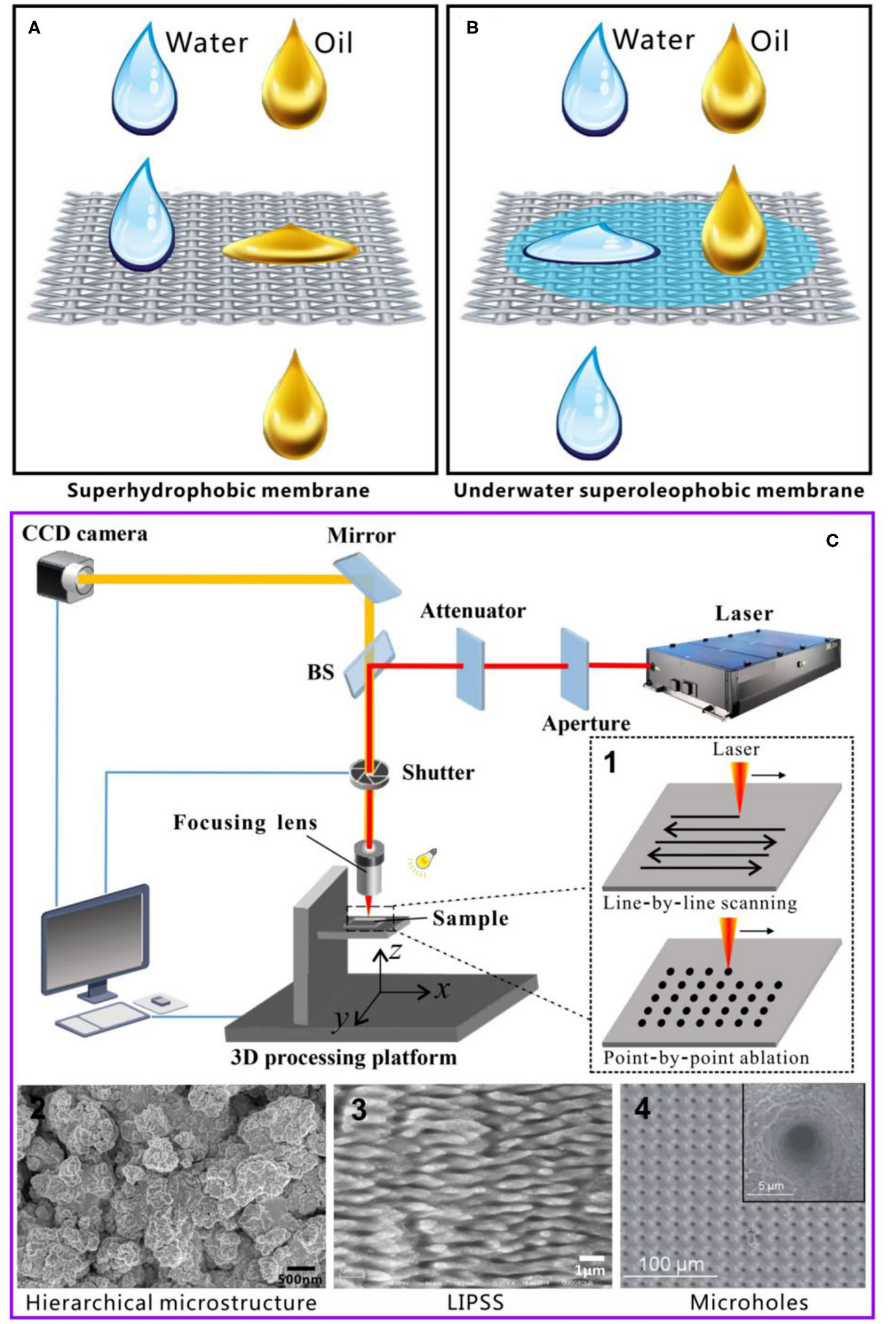
**(A)** Schematic of OWS by the superhydrophobic porous materials through an “oil-removing” manner. **(B)** Schematic of OWS by the underwater superoleophobic porous materials through a “water-removing” manner. **(C)** Schematic of a laser processing system. First inset in **(C)**: two typical manners (line-by-line scanning and point-by-point drilling) in laser processing. Second to fourth insets in **(C)**: examples of the typical microstructures induced by femtosecond laser. Reproduced from Yong et al. (2017b, 2019a,b) with the permission of Yong et al. Reproduced from Li G. et al. (2016) with permission of the Royal Society of Chemistry.

Based on the different interfacial effects of water and oil, superwetting porous materials with completely opposite wettability to oil and water are used in OWS in recent years (Xue et al., 2014; Chu et al., 2015; Wang et al., 2015; Yong et al., 2016a; Gupta et al., 2017; Yong J. et al., 2018b). The superwetting porous materials have either superhydrophobicity/superoleophilicity or superhydrophilicity/underwater superoleophobicity. In 2004, Feng et al. (2004) prepared a superhydrophobic and superoleophilic metal mesh coated with polytetrafluoroethylene (PTFE). When the OWM was poured on such a mesh, the water in the OWM was repelled and stayed on the mesh due to the superhydrophobicity of the mesh. In contrast, the superoleophilicity allowed the oil to wet and pass through the metal mesh, thereby achieving OWS (Figure 1A). Such superhydrophobic porous materials are often called as “oil-removing” materials. In 2011, Xue et al. (2011) achieved OWS by using an underwater superoleophobic hydrogel-coated metal mesh. The composite mesh presented superhydrophilicity in the air but superoleophobicity underwater. Once an OWM was poured on the water-pre-wetted mesh, the water in the OWM would penetrate the mesh. In contrast, the oil remained above the mesh because of the underwater superoleophobicity (Figure 1B). This kind of underwater superoleophobic porous membranes is termed as “water-removing” separation materials. Based on the above separation principle, more and more superwetting porous materials have been developed for achieving OWS (Xue et al., 2014; Chu et al., 2015; Wang et al., 2015; Yong et al., 2016a; Gupta et al., 2017; Yong J. et al., 2018b).

## Features of the Femtosecond Laser Processing

The femtosecond laser has become one of the most advanced tools in the field of micro/nano-fabrication because of its extremely short pulse width and ultrahigh peak power density (Vorobyev and Guo, 2013; Sugioka and Cheng, 2014a; Yong et al., 2019b; Bai et al., 2020). Femtosecond laser processing possesses many unique features, such as small heat-affected area, high spatial resolution, a wide range of processable materials, and non-contact processing (Sugioka and Cheng, 2014b; Yong et al., 2017b; Zhang and Sugioka, 2019). A femtosecond laser can process almost any given materials and can directly produce micro- and nano-scale structures on the material surface by simple one-step ablation. Figure 1C shows a typical femtosecond laser micromachining system. The femtosecond laser pulses generated by the laser system are focused on the material surface by a lens and induce the required microstructures on the material surface through the interaction between the ultrafast laser pulses and the materials. Generally, there are two typical manners in laser processing: line-by-line (progressive) scanning and point-by-point drilling (ablation) (first inset of Figure 1C). The former manner can produce uniform rough microstructures on the material surfaces, such as the micro/nanoscale hierarchical microstructures (e.g., second inset of Figure 1C; Yong et al., 2017b) and the typical laser-induced period surface structure (LIPSS) (e.g., third inset of Figure 1C; Yong et al., 2019a). The femtosecond laser-induced microstructures have high roughness and greatly increase the actual surface area of the substrates. The latter manner can be used to generate holes array structure (e.g., fourth inset of Figure 1C; Li G. et al., 2016). The laser-drilled through holes are funnel-shaped and their diameter can be as small as several micrometers and even sub-micrometer. Both the inner wall and the rim of the microholes are decorated with micro/nanostructures which can allow the microholes to have superwettability.

Surface wettability mainly depends on the morphology and chemical composition for a solid substrate (Tian et al., 2014; Wen et al., 2015; Sun et al., 2016; Yong et al., 2017d). The features of femtosecond laser micromachining enable this technology to successfully control the wettability of solid materials (Chen et al., 2013; Yong et al., 2015c; Yong J. et al., 2018a). The femtosecond laser-based method shows some advantages over traditional methods in achieving extreme wettability. (a) The femtosecond laser can ablate most materials, independent of special substrates. (b) Hierarchical micro/nanostructures can form on the material surface directly only through a single-step ablation. (c) The laser-processed position can be precisely moved by the control program. A variety of micro-pattern structures can be flexibly obtained without an expensive mask to realize complex and fine control of surface wettability. (d) Laser treatment is a mechanical and physical process without involving toxic or dangerous chemical reactions or operations.

Based on these advantages, a series of extreme wetting characteristics have been achieved through femtosecond laser micromachining, such as superhydrophobicity (Baldacchini et al., 2006; Zhang et al., 2012; Yong et al., 2013a,b, 2014b), underwater superoleophobicity (Yong et al., 2014a, 2015a,b), underwater superaerophobicity (Yong et al., 2017a; Yong J.L. et al., 2018), super-slippery property (Yong et al., 2017c; Yong J. et al., 2018c), underwater superpolymphobicity (Yong et al., 2019a,c,d), supermetalphobicity (Yong et al., 2020b; Zhang et al., 2020), and so on. For example, superhydrophobicity usually results from the combination of surface microstructure and the low-surface-energy chemical composition, which can be obtained by directly generating rough microstructures on a hydrophobic substrate through laser ablation or modifying the laser-structured hydrophilic substrate with low-surface-energy molecules (Zhang et al., 2012; Yong et al., 2015c). By contrast, underwater superoleophobicity usually results from the combination of surface microstructure and the high-surface-energy chemical composition, which can be achieved on the inherently hydrophilic substrate after the formation of rough microstructure by laser ablation (Yong et al., 2017d; Yong J. et al., 2018a). The diversity of the obtained superwettability endows the laser-structured superwetting porous materials with the OWS ability in a variety of manners.

## Oil/Water Separation Based on the Femtosecond Laser-Structured Porous Superwetting Materials

### Formation of Superhydrophobic Microstructures on a Porous Substrate

**Figure 2:**
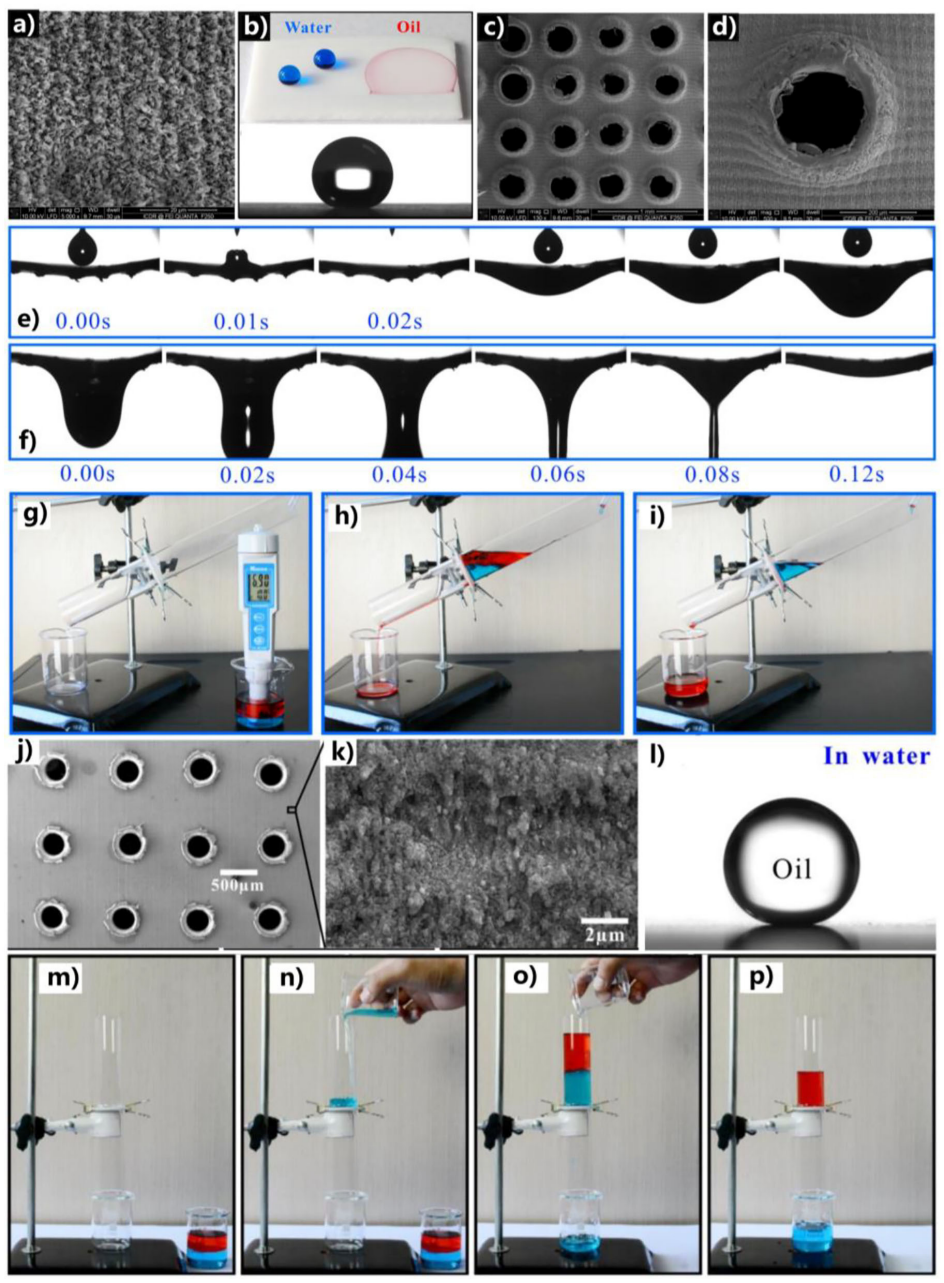
OWS by using the porous superhydrophobic or underwater superoleophobic sheet structured by femtosecond laser. **(a)** SEM image of the laser-induced rough structure on a PTFE substrate. **(b)** Superhydrophobicity and superoleophilicity of the laser-structured PTFE sheet. Blue liquid and the below droplet: water droplets; red liquid: oil droplet. **(c,d)** SEM images of the laser-structured PTFE sheet with through microholes array. **(e)** Process of releasing oil droplets on the laser-structured porous PTFE sheet. **(f)** Oil permeating the PTFE sheet. Separating the mixture of oil (red) and water (blue) by the superhydrophobic porous PTFE sheet: **(g)** before starting, **(h)** adding the OWM into the designed separation device, and **(i)** after separation. **(j)** SEM image of the laser-structured iron sheet with through microholes array. **(k)** SEM image of the microstructure induced by laser on an iron substrate. **(l)** Oil droplet on the laser-ablated iron sheet in water. Separating the mixture of oil (red) and water (blue) by using the porous laser-structured iron sheet: **(m)** before starting, **(n)** prewetting the sheet by water, **(o)** adding the OWM into the designed separation device, and **(p)** after separation. Reproduced from Yong et al. (2016b) with the permission of the Elsevier B.V. Reproduced from Yong et al. (2020a) with the permission of Yong et al.

Yong et al. (2017b) directly constructed a micro/nanoscale hierarchical structure on an intrinsically hydrophobic PTFE surface by femtosecond laser ablation (Figure 2a). Water droplets on the laser-ablated surface have a contact angle (CA) of 155.5°, while oil droplets rapidly spread on such surface with a final CA of ~0° (Figure 2b). It indicates that the rough PTFE surface has both superhydrophobicity and superoleophilicity. An array of perforated holes with a diameter of ~260 μm was further formed on the laser-ablated PTFE sheet (thickness = 0.3 mm) through a mechanical drilling processing (Figure 2c). The rough structure remains intact around the microholes (Figure 2d). The backlight could pass through the microholes but was blocked by the rest region when observed by optical microscopy, demonstrating the drilled holes are throughout the sheet. When oil droplets were gradually dropped on the perforated rough sheet, they quickly spread out because of the superoleophilicity. The oil further permeated through the sheet along the microholes (Figure 2e) and eventually dropped down (Figure 2f). By using the porous superhydrophobic PTFE sheet as a separation membrane, OWM can be efficiently separated. A manually simple separation device was prepared by sandwiching the superhydrophobic porous sheet between two tubes (Figure 2g). When an immiscible OWM was feed into the designed separation device, oil completely permeated through the separation membrane, while the water was prevented and remained above the PTFE sheet (Figure 2h). Finally, the mixture was successfully separated (Figure 2i). Furthermore, such a device can also separate the OWMs composed of strong acid/alkali solutions.

### Formation of Superhydrophobic Microholes on a Membrane

Zhang et al. (2017) prepared an array of microholes on an aluminum foil via femtosecond laser drilling. The microhole diameter could be tuned from 14.1 to 31.3 μm by adjusting the energy and number of laser pulses. The microholes were surrounded by microscale fragments with a size of 2–10 μm. The inner wall of the microholes was also covered with micro/nanoscale wrinkles. After the further fluoroalkylsilane modification and ablating lower side by femtosecond laser, the upper side of the foil shows superhydrophobicity with a CA of 158.4° to a water droplet, while the lower side is superhydrophilicity. A simple oil-absorption barrel was prepared by curling the superwetting aluminum foil. The barrel could remove oil from the water surface. When the barrel was partly immersed in a beaker filled with water and oil (octane), oil rapidly entered into the barrel but the water was prevented from entering into the barrel because of the superhydrophobicity. Finally, the spilled oil was completely collected by the barrel.

### Formation of Underwater Superoleophobic Microstructures on a Porous Substrate

Different from superhydrophobicity, underwater superoleophobicity can also be obtained on many materials (such as various metal substrates) by femtosecond laser treatment (Yong et al., 2015c). Yong et al. (2020a) prepared a porous underwater superoleophobic iron sheet. An array of through microholes with a diameter of about 300 μm was generated on the metal sheet through a mechanical drilling process in advance (Figure 2j). Then, the whole surface of the sheet was ablated by the femtosecond laser to create rough laser-induced nanoripples structure on the surface of the porous iron sheet (Figure 2k). The sheet thus showed quasi-superhydrophilicity and it could be fully wetted by water. When immersed in water, oil droplets on such a sheet have a CA of 164° (Figure 2l) and could easily roll away, revealing great underwater superoleophobicity of the structured porous metal sheet. The combination of the underwater superoleophobicity and the through microholes enables the femtosecond laser-structured porous iron sheet to separate OWM through the “water-removing” manner. Before separation (Figure 2m), the porous sheet needed to be pre-wet by little water (Figure 2n). When the OWM was poured onto the porous sheet, the water in the OWM could easily permeate the sheet (Figure 2o). On the contrary, the superoleophobicity (underwater) prevented oil from passing through the separation membrane, so that the oil always remained above the porous sheet (Figure 2p). In the end, the OWM was completely separated, and the femtosecond laser treatment can endow a wide range of metal sheets with OWS capacity (Yong et al., 2015c, 2020a).

Yin et al. (2017) fabricated nanostructures on a stainless steel mesh (300 mesh) through femtosecond laser ablation. Uniform periodic nanoripples with a width of 500–800 nm and a depth of 130 nm were induced on the surface of the mesh wire. The formation of the nanoripple structures changed the mesh to superhydrophilic in air. When immersed in water, the mesh exhibited ultralow oil-adhesive superoleophobicity. The mesh pores could serve as the penetrable microholes for a separation. The superhydrophilic and underwater superoleophobic properties endow the structured mesh with the OWS capacity with high efficiency above 99%. Yang et al. (2019) used femtosecond laser direct writing to prepare a porous titanium foam whose surface was covered by numerous nanoripples and nanoparticle structures. The resultant titanium foam is rich in porous structures and exhibits superhydrophilicity and underwater superoleophobicity. An oil-in-water emulsion was successfully separated by the foam.

### Formation of Underwater Superoleophobic Microholes on a Membrane

Li G. et al. (2016) prepared uniform micropores on an aluminum foil through the femtosecond laser perforating process. The diameter of the pores could be adjusted from 2.4 to 32 μm. Fine nanostructures cover on the inside wall and the rim of the generated micropores. The rough porous aluminum foil is superhydrophilic (water CA = 7.8°) and underwater superoleophobic (oil CA = 153.1°). The as-prepared porous foil was successfully used to separate the OWM by a “water-removing” manner. Ye et al. (2016) prepared funnel-shaped microholes with a diameter of about 55 μm on a titanium foil via femtosecond laser micro-drilling. The drilling treatment also produced rich irregular nanoscale protrusions on the wall of every microhole. The microhole-structured foil exhibited underwater superoleophobicity with a CA of 159.6° to an underwater oil droplet. When the OWM consisted of sesame oil was poured on the porous foil prewetted by water, the oil in the OWM stayed above the foil while the water could completely permeate through the filter.

### Discussion

Each approach has its benefits and drawbacks. For the abovementioned method of generating superwetting surface microstructures on a porous substrate, various kinds of materials can be used as the substrates, such as metal mesh, polymer mesh, foam, sponge, etc. The thickness of the adopted porous substrate can be large enough to ensure a high mechanical strength of the as-prepared separation materials. However, the diameter of the pores, which depends on the adopted substrate, cannot be artificially designed. Although a large pore can provide a high liquid flux in OWS, it also results in a low intrusion pressure. Low intrusion pressure sometimes will lead to a failed separation. The pore diameter of these substrates is usually larger than 10 μm. For the abovementioned method of generating superwetting microholes, the diameter of the femtosecond laser-drilled holes can be simply adjusted by using different laser and processing parameters and it can be as small as several micrometers and even smaller. However, the adopted substrate must be thin membrane or sheet (usually with the thickness smaller than 0.1 μm), otherwise, the laser pulses cannot burn through the membrane. The substrate is so thin that the as-prepared separation materials maybe face mechanical strength problems. Therefore, we should choose the most appropriate strategy to obtain the OWS materials by femtosecond laser processing according to the specific application situations and the substrate materials, as well as the advantages and disadvantages of different types of the laser-designed separation materials.

In comparison to other methods to prepare superwetting separation materials, femtosecond laser processing has unique advantages. For example, it can process almost any given materials and then endow those materials with superwettability for OWS. Besides, such technology can generate not only superwetting surface microstructures but also through microholes. Both surface superwettability and microholes structure play a crucial role in the process of OWS. However, such technology still suffers from itself limitations, such as low processing efficiency and the weak ability of processing non-planar surface, toward large-scale OWS application. These technical limitations should be gradually solved in practical application.

## Conclusions and Outlook

Frequent oil leakage accidents and illegal discharge of industrial oily wastewater not only cause enormous economic losses but also seriously damaged the ecological environment. Therefore, the research and development of efficient OWS materials are of great significance to the healthy development of human society. This paper reviews the current progress of achieving OWS by using femtosecond laser-induced superwetting materials, including superhydrophobic “oil-removing” porous sheets and underwater superoleophobic “water-removing” porous sheets. Compared to common methods to prepare superwetting separation materials, the femtosecond laser can process a wide range of materials and can simply prepare superwetting microstructure by a single-step ablation. Apart from the induced different superwettabilities, the femtosecond laser can also produce microhole structures on a thin membrane. The combination of the laser-induced superwettability and microholes enables the femtosecond laser-structured materials to separate OWMs through various manners.

Although these laser-structured materials show significant potential in the field of OWS, there are still many challenges before they can practically solve the problem of oil pollution. Firstly, the fabrication efficiency of separation materials by femtosecond laser should be improved toward large-scale practical applications. Secondly, the durability of the superhydrophobicity and superoleophobicity of the laser-structured materials should be considered which is important to the service life of the designed separation device. Thirdly, real oil contaminants are usually very complex and diverse in comparison to the pure oily liquids in the lab. Currently, the superwetting porous materials can separate low-viscosity OWMs but are difficult to effectively separate the mixture of high-viscosity oils and water. The purification of high-viscosity crude oil pollution is still an unsolved worldwide problem. Finally, a practical large separation instrument needs to be designed to practically prevent the environmental pollution caused by spilled oil and oily wastewater. We believe that the advantages of the femtosecond laser enable the structured superwetting materials to have an exciting future in OWS applications.

## Author Contributions

NF wrote the manuscript. JY contributed to significant discussions and revised the paper. All authors contributed to the article and approved the submitted version.

## Conflict of Interest

The authors declare that the research was conducted in the absence of any commercial or financial relationships that could be construed as a potential conflict of interest.

## Footnotes

^1^https://en.wikipedia.org/wiki/Exxon_Valdez_oil_spill

^2^https://en.wikipedia.org/wiki/Prestige_oil_spill

^3^https://en.wikipedia.org/wiki/Deepwater_Horizon_oil_spill
